# Determinants of knowledge and attitudes toward Mpox among medical students: A cross-sectional study from Kurdistan region of Iraq

**DOI:** 10.1371/journal.pone.0350502

**Published:** 2026-05-29

**Authors:** Ibrahim A. Naqid, Ahmed A. Mosa, Nawfal R. Hussein, Iman Haji Yaseen, Kareen Yarwant Naisan, Israa Taher Shuker, Sidra Anwar Kadhim, Aveen Saleem Shareef, Mina A. Almohammed

**Affiliations:** 1 Department of Biomedical Sciences, College of Medicine, University of Zakho, Zakho, Kurdistan Region of Iraq; 2 Department of Clinical Sciences, College of Medicine, University of Zakho, Zakho, Kurdistan Region of Iraq; 3 Director General of Health-Duhok Governorate, Ministry of Health, Duhok, Kurdistan Region of Iraq; Federal University Otuoke, NIGERIA

## Abstract

**Introduction and Objectives:**

Mpox is a re-emerging zoonotic viral infection that has recently caused widespread outbreaks in previously non-endemic regions, becoming an international public health concern. Medical students are future healthcare workers (HCWs); they play a critical role in public health initiatives and targeted preventive measures. Therefore, it’s necessary to assess and enhance their knowledge and attitude concerning new emerging infectious diseases like Mpox. Therefore, in this study, we aimed to fill the literature gap by assessing medical students’ knowledge and attitudes regarding Mpox infection in the Kurdistan Region of Iraq. Additionally, the study intends to demonstrate a correlation between knowledge and attitudes levels and demographic characteristics.

**Materials and Methods:**

This cross-sectional study was carried out in Duhok Province, Kurdistan Region, Iraq, from January 30, 2024, to April 4, 2024, involving a total of 330 medical students. Data were collected using a structured self-administered questionnaire comprising 36 items categorized into three distinct sections: demographic characteristics, knowledge, and attitude. To maximize the response rate, both digital questionnaires distributed via Google Forms and traditional paper-based formats were employed.

**Results:**

The participants’ mean age was 21.57 ± 1.69 years, with a slight female predominance (52.12%). Only (8.18%) of students had received Mpox training program. Social media was the primary information source for (36.97%) of students. The median [IQR] scores for knowledge and attitude were 20 [16 –23], and 42 [37 –45], respectively. About (40.91%) of respondents exhibited good knowledge, while (47.88%) demonstrated a positive attitude. A better knowledge score was significantly associated with age (p = 0.0067), and prior awareness of smallpox (p = 0.0011). In contrast, students who relied on friends as the main source of Mpox information demonstrated significantly lower knowledge levels (p = 0.015). Attitude was significantly linked to gender (p = 0.012), and a history of chickenpox (p = 0.0019).

**Conclusion:**

Medical students in the present study demonstrated relatively insufficient knowledge about Mpox. However, their attitudes were more favorable, albeit still suboptimal. These findings highlight the urgent necessity for structured educational programs on emerging infectious diseases for health sciences students, with prioritization of students lacking knowledge of smallpox and chickenpox, and those who depended on non-formal information sources. Such programs are essential to equip students with the necessary knowledge and skills to enhance their ability to respond to and control infectious diseases outbreaks.

## Introduction

Mpox is a re-emerging zoonotic viral disease caused by the Mpox virus, a double-stranded DNA virus belonging to the genus *Orthopoxvirus* within the *Poxviridae* family [1]. Smallpox and Mpox belong to the same family, causing comparable symptoms, which complicates the differentiation between both illnesses [2]. The discovery of the Mpox virus dates back to 1958, when it was identified in the Danish Laboratory in monkey colonies [3]. However, the first human Mpox case was reported in the Democratic Republic of Congo (DRC) in 1970. Subsequently, West and Central Africa became endemic regions due to the widespread transmission of the infection [4]. In 2003, the first case of Mpox outside endemic regions was reported in the United States [5]. On July 23, 2022, due to the rapid rise of Mpox cases outside the endemic areas, the World Health Organization (WHO) announced Mpox infection as a public health emergency of international concern [6].

Humans acquire infection mainly through contact with respiratory droplets, contaminated surfaces, skin lesions, and body fluids. Additionally, rodents, dogs, and squirrels are potential contributors to the transmission of Mpox to humans [7,8]. The disease onset is characterized by a prodrome of fever in the early days, with other associated symptoms such as headache, fatigue, myalgia, back pain, and chills [9,10]. The condition is typically diagnosed with the appearance of well-circumscribed skin rashes of various sizes that initially appear on the face, followed by centrifugal distribution. In Mpox, lymphadenopathy may develop before or during the course of skin rashes, which is a distinguishing feature from smallpox [11,12]. Although Mpox is a self-limiting infection. Nonetheless, severe manifestations have been reported in immunocompromised patients, pregnant women, and the pediatric age group [13].

Rapid application of preventive measures, such as early identification and management, is crucial, especially in the context of infectious diseases. Nevertheless, lack of knowledge, inappropriate attitude, and unwillingness to adopt recommended guidelines are major challenges in the prevention of Mpox re-emergence. This may negatively impact control programs and public health awareness [11]. It is imperative to equip health sciences students and healthcare workers (HCWs) with the necessary knowledge regarding the diagnosis, treatment, and prevention of infection, as this will facilitate early case identification and contribute to improved outcomes [14]. In this respect, previous studies revealed that HCWs and health sciences students have an insufficient understanding of Mpox infection [15–17]. These findings highlight the crucial need to boost the students’ understanding of the Mpox infection.

Medical students are future HCWs; they play a critical role in public health initiatives and targeted preventive measures. Therefore, it’s necessary to assess and strengthen medical students’ knowledge and attitudes concerning new emerging infectious diseases like Mpox. Because they can positively influence public perception of diseases, enhance community awareness, and promote effective preventive practices. Globally, several studies have been carried out to assess medical students’ knowledge and attitudes regarding Mpox [11,16–18]. However, to the best of our knowledge, no studies among medical students regarding this topic have been conducted in the Kurdistan Region of Iraq. Hence, the current study aimed to fill this geographical gap in the literature by evaluating medical students’ knowledge and attitudes regarding Mpox infection in Iraqi Kurdistan. Additionally, the study intends to demonstrate a correlation between knowledge and attitudes levels and demographic characteristics. The study findings are expected to support the development of targeted educational initiatives for medical students in the region and contribute to broader efforts of combating Mpox misconceptions and strengthening regional preparedness for this infection.

## Materials and methods

### Study design and participants

This cross-sectional study was carried out in Duhok Province, Kurdistan Region of Iraq, targeting medical students from Zakho and Duhok Medical Colleges. Between January 30, 2024, to April 4, 2024, data were collected using a structured self-administered questionnaire. To maximize the response rate, both digital questionnaires distributed via Google Forms and traditional paper-based formats were employed. A total of 330 medical students participated in this study. This sample size is sufficient to represent the targeted population, which consisted of 1338 medical students enrolled in Zakho and Duhok Medical Colleges, with a 95% confidence interval, 50% response distribution, and a 5% margin of error. The sample size was determined using an online calculator available at Raosoft (http://www.raosoft.com/samplesize.html). The study design and methods were developed in alignment with the Standards for Strengthening the Reporting of Observational Studies in Epidemiology (STROBE) guidelines.

### Instrument development

The initial questionnaire employed in this study was adapted from a previously published and validated instrument designed to assess medical students’ knowledge and attitudes regarding Mpox infection. [11]. This questionnaire was subsequently reviewed and evaluated by the formed expert panel, consisted of three investigators (an infectious disease specialist, a microbiologist, and a research methodologist) to ensure clarity, comprehensive coverage of all key aspects, and to establish face validity, content validity, and cross-cultural adaptation. Initially, these experts independently reviewed the questionnaire and rated each item on a 4-point relevance scale, ranging from 1 (not relevant) to 4 (highly relevant). Then after, the expert ratings were aggregated to calculate item-level content validity index (I-CVI) and scale-level content validity index/average (S-CVI/Ave). For calculation of these indexes, ratings of 3 or 4 were recoded as 1 (indicating relevance), whereas ratings of 1 or 2 were recoded as 0 (indicating non-relevance). All items in the knowledge and attitude sections achieved an I-CVI and S-CVI/Ave of 1, indicating that each item was judged as either quite relevant or highly relevant by all experts. Furthermore, the formal language of teaching in Medical Colleges in Kurdistan Region of Iraq is English. Since, all students possess adequate English proficiency, the questionnaire was administered in English language; therefore, translation and back-translation were not required. However, certain vocabularies were highlighted by the expert panel in order to evaluate students understanding of these terms, with particular emphasis to include students from all stages of training (pre-clinical and clinical) and diverse level of English language proficiency.

### Pilot testing

These experts also trained five authors prior to data collection to ensure standard and accurate administration of questionnaires. Furthermore, the trained authors were invited by expert panel to provide feedback on the questionnaire in terms of clarity, readability, wording, overall comprehensibility, and potential highlighted vocabularies. A pilot test was then carried out among 20 medical students from different academic stages by the trained research members to ensure clarity, comprehensiveness, and the time required for completion. The students recruited for pilot testing were also asked to provide feedback on any items they found confusing or difficult to understand, which could impact the overall quality of the questionnaire. No major feedback regarding clarity emerged from the pilot testing, and students demonstrated good overall comprehension including well-understanding of highlighted vocabularies. In addition, no any specific suggestions were made to add or remove any items. Therefore, no any modifications were made following the pilot testing. Internal consistency was assessed using Cronbach’s alpha, which yielded satisfactory results. The Cronbach’s alpha for the knowledge and attitude sections were 0.72, and 0.71, respectively.

### Study tool

The final study questionnaire, comprising 36 items was categorized into three distinct sections;

#### Section I – Basic demographic features.

The first section was designed to obtain demographic information, including age, gender, place of residence, and year of study. It also assessed students’ knowledge related to smallpox, history of COVID-19 and influenza vaccination, personal history of chickenpox infection, and whether they had received any Mpox-related training programs. This section concluded by identifying the sources that students utilized to acquire knowledge about Mpox.

#### Section II – Knowledge.

This section consisted of 14 items to assess medical students’ knowledge related to Mpox, and students were required to classify their understanding of each statement as either “yes”, “no”, or “uncertain”. These statements covered a spectrum of different aspects of Mpox, including the responsible pathogen, its potential as a re-emerging disease, transmission methods, clinical manifestations, and preventive strategies. Additionally, it addresses misconceptions, such as the purported link between Mpox outbreaks and homosexuality, and the availability of any licensed vaccine for Mpox infection. The response to knowledge section items were scored as follows: two for “yes”, one for “uncertain”, and zero for “no”. This gave the knowledge section a maximum score of 28, with greater scores indicating better knowledge about the disease. However, the sixth item in the knowledge section, “Mpox outbreaks in 2022 were noted to be related to homosexuality” was reversely-scored.

#### Section III – Attitude.

The final section, comprising 12 items, explores medical students’ attitudes toward Mpox infection. In this section, a five-point Likert scale ranging from 5 to 1, from strongly agree to strongly disagree, was utilized to measure response to each question. A potential score in the attitude section ranged from 12 to 60; the greater the value, the more positive the attitude. Nonetheless, in this section, the negative attitude items were reversely-scored to maintain coherence with positively expressed statements.

#### Scoring categorization method.

Participants’ knowledge and attitude were categorized using the median value as the cutoff. Those who scored above the median (>20) and (>42) were classified as having good knowledge and a positive attitude, respectively; while students who scored at or below the median (≤20) and (≤42) were considered to have poor knowledge and a negative attitude, respectively. The rationale for considering median values as a cutoff for categorization was based on following points. First, no validated cutoffs exist for Mpox knowledge, and attitude constructs among our population. Secondly, the vast majority of our study participants had not received a formal education and/or training about Mpox; therefore, applying a strict 80% cut-off (bloom’s criteria) which is mostly applied among trained individuals to define good knowledge, and a positive attitude, could underestimate adequate knowledge, and a positive attitude in our context. Additionally, the median split was data-driven and sample-appropriate, producing balanced and interpretable groups for exploring statistical associations between independent and dependent variables using logistic regression analyses. Finally. this criterion has been widely adopted in KAP studies  [11,19].

### Inclusion and exclusion criteria

The inclusion criteria were restricted to participants who were students in one of the chosen Medical Colleges, aged more than eighteen years, and who voluntarily consented to be enrolled in the study. Conversely, dental and pharmacy students, students who did not provide informed consent, and surveys with incomplete responses or missing data were excluded from the study.

### Ethical consideration

The final study questionnaire was granted approval on January 22, 2024, with a reference number (JAN2024/UOZEE40) by the Ethics and Scientific Committee of the College of Medicine, University of Zakho, Iraqi Kurdistan. Before recruitment, all students provided written informed consent. Data were collected anonymously without recording any identifiable student information to ensure confidentiality and anonymity. All data were exclusively used for the purposes of this study. Ethical Standards for Medical Research Involving Human Subjects, in accordance with the Declaration of Helsinki, were rigorously followed.

### Statistical analysis

GraphPad Prism version 10.5 and Microsoft Excel were employed to manage, clean, and analyze the collected data. Descriptive variables of the participants were summarized as percentages and frequencies, whereas continuous variables were translated into means and standard deviations. The sum of knowledge and attitude scores was summarized into means, standard deviations, medians, and interquartile ranges [IQR]. The Kolmogorov-Smirnov test revealed a non-normal distribution of continuous dependent variables. Therefore, non-parametric tests were employed. The statistical association between demographic variables and knowledge and attitude scores was evaluated using the Chi-square test for categorial variables and two-tailed Mann-Whitney test for continuous variables. Predictors of knowledge and attitudes were identified by calculating adjusted odds ratios (aORs) with 95% confidence intervals (CIs) using a multivariable binary logistic regression model. Additionally, sensitivity analyses were performed to ensure robustness of the results by modeling dependent variables as continuous measures and applying alternative cut-off criteria. Detailed methods and results of the sensitivity analyses are provided in Supplementary Material 1. A p-value of less than 0.05 was considered a statistically significant association.

## Results

### Demographic characteristics

This study recruited 330 medical students. The participants’ mean age was 21.57 ± 1.69 years. The gender distribution was approximately equal. Students in the clinical stages represented about two-thirds (61.52%) of the study sample. About two-thirds (62.12%) of participants were familiar with smallpox. Among the students, (63.3%) had received the COVID-19 vaccine, and only 27 students had received Mpox training program. **Table 1** shows demographic features and vaccination status of the study participants.

**Table 1:** Demographic characteristics and vaccination status of the students (n = 330).

Variables	n (%)
**Age (Years)**	
21 and below	160 (48.48)
22 and above	170 (51.52)
Mean (SD)	21.57 (1.69)
**Gender**	
Male	158 (47.88)
Female	172 (52.12)
**Stage**	
Pre-clinical stages	127 (38.48)
Clinical stages	203 (61.52)
**Place of Residence**	
Urban/City	304 (92.12)
Rural	26 (7.88)
**Knowledge of smallpox**	
Yes	205 (62.12)
No	125 (37.88)
**Vaccinated against COVID-19**	
Yes	209 (63.3)
No	121 (36.67)
**Received seasonal influenza vaccine**	
Yes	24 (7.27)
No	306 (92.73)
**History of chickenpox disease**	
Yes	201 (60.90)
No	129 (39.10)
**Received training programs about Mpox**	
Yes	27 (8.18)
No	303 (91.82)

### Sources of information about Mpox virus

**Fig 1:**
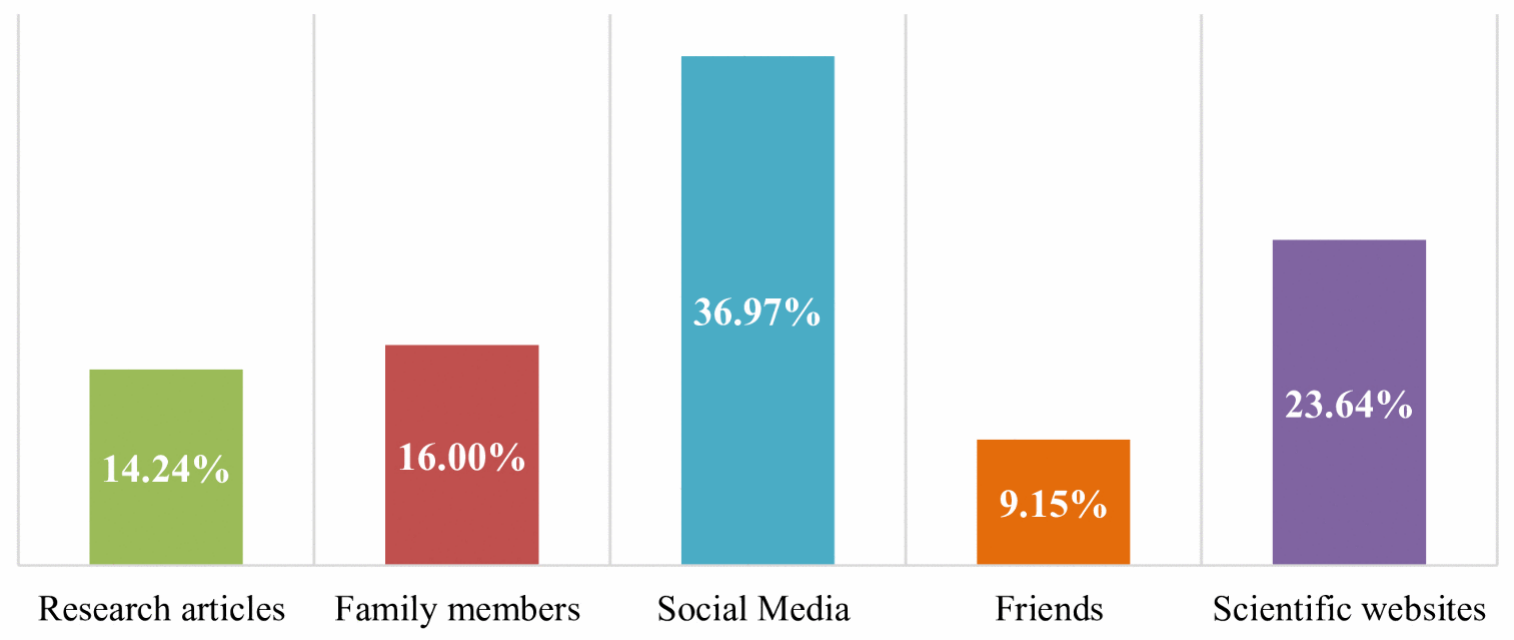
Sources of Mpox knowledge among students (n = 330).

Fig 1 illustrates sources of Mpox knowledge. The primary source of information for about one-third (36.97%) of the students was social media. Around one quarter of students reported family and friends as their source of information. Lastly, (23.64%), and (14.24%) of students utilized scientific websites and research articles, respectively, as sources of knowledge.

### Knowledge about Mpox infection

**Fig 2:**
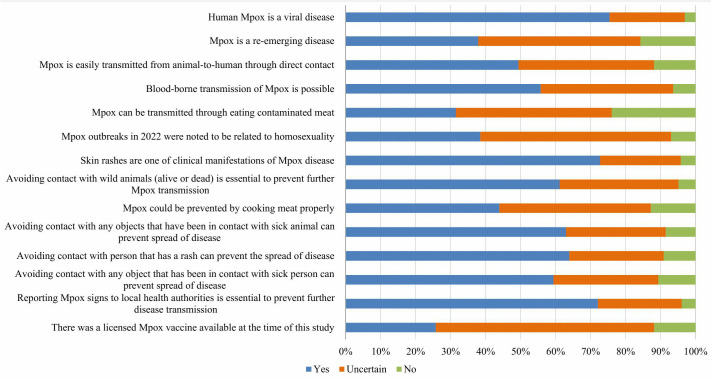
Knowledge of medical students about Mpox infection (n = 330).

Fig 2 and S2 Table 2A summarize participants’ knowledge of Mpox infection. Approximately three-quarters of students (75.45%) accurately recognized Mpox as a viral disease, while less than two-fifths (37.88%) knew that Mpox is considered a re-emerging infection. Generally, students demonstrated a moderate understanding of routes of transmission. However, awareness of transmission through contaminated meat was considerably low. Notably, (38.48%) of students demonstrated a misconception by associating Mpox outbreaks with homosexuality. With respect to preventive measures (Q8-Q12), students’ answers were mostly comparable for all of the present items. Nevertheless, a notably lower proportion (43.94%) recognized proper cooking of meat as a preventive measure. Additionally, (72.12%) of the sample were aware of the necessity of reporting suspected Mpox cases to local health authorities, while only (25.76%) were aware of the availability of a licensed vaccine.

### Attitude towards the Mpox infection

**Fig 3:**
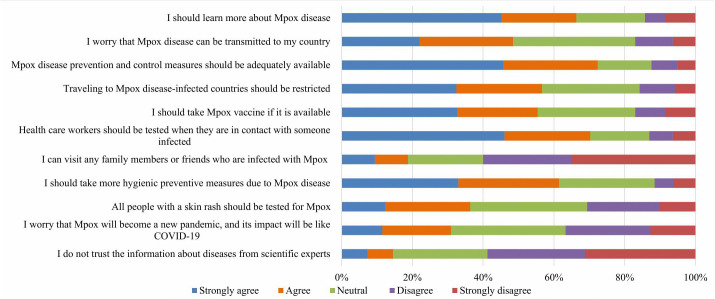
Medical students’ attitudes toward Mpox infection (n = 330).

About two-thirds of study participants either strongly agreed or agreed with the statements that “they should learn more about Mpox infection”, “that adequate preventive and control measures should be available”, and “that HCWs should be tested following contact with infected individuals”. Moreover, (36.36%) of participants expressed concern that Mpox could potentially lead to a new pandemic similar to COVID-19, while (19.39%) believed that the disease might be linked to efforts to reduce the global population. Notably, only (14.54%) of respondents expressed distrust in information from scientific experts. Fig 3 and S2 Table 2B provide a detailed summary of students’ attitudes toward Mpox infection.

### Association between demographic characteristics and knowledge and attitude about Mpox

Table 2 illustrates students’ mean and median values for both the knowledge and attitude sections. Good knowledge of Mpox was demonstrated by (40.91%) of respondents, while a positive attitude was noted in (47.88%) of students. Table 3 displays the associations between knowledge, and attitude scores and demographic features. Knowledge score was significantly associated with students’ mean age (p = 0.0067), awareness of smallpox (p = 0.0011). In contrast, students who relied on friends as the main source of Mpox information demonstrated significantly lower knowledge levels (p = 0.015). However, no other demographic variables showed a statistically significant association. Regarding students’ attitudes, significant associations were observed with gender (p = 0.012), and a history of chickenpox infection (p = 0.0019), while the remaining variables did not show statistically significant associations.

**Table 2:** Summation of knowledge, and attitude score distribution (n = 330).

	Knowledge sum	Attitude sum	Total score
**Mean (SD)**	19.54 (±4.09)	41.02 (±5.81)	60.56 (±7.68)
**Median [IQR]**	20 [16 –23]	42 [37 –45]	61 [55-66]

**Table 3:** Association between students’ demographic characteristics and their knowledge and attitudes toward Mpox infection (n = 330).

Variables	Knowledge		p-value	Attitude		p-value
	Good (>20) n (%)	Poor (≤20) n (%)		Positive (>42) n (%)	Negative (≤42) n (%)	
**Age (Years)**						
21 and below	57 (35.63)	103 (64.37)	0.058	74 (46.25)	86 (53.75)	0.57
22 and above	78 (45.88)	92 (54.12)		84 (49.41)	86 (50.59)	
Mean (SD)	21. 86 (±1.75)	21.35 (±1.65)	0.0067*	21.59 (±1.54)	21.53 (±1.85)	0.49*
**Gender**						
Male	63 (39.87)	95 (60.13)	0.71	87 (55.06)	71 (44.94)	0.012
Female	72 (41.86)	100 (58.14)		71 (41.28)	101 (58.72)	
**Stage**						
Pre-clinical stages	44 (34.65)	83 (65.35)	0.067	55 (43.31)	72 (56.69)	0.19
Clinical stages	91 (44.83)	112 (55.17)		103 (50.74)	100 (49.26)	
**Place of Residence**						
Urban/City	124 (40.79)	180 (59.21)	0.88	150 (49.34)	154 (50.66)	0.069
Rural	11 (42.31)	15 (57.69)		8 (30.77)	18 (69.23)	
**Knowledge of smallpox**						
Yes	98 (47.80)	107 (52.20)	0.0011	104 (50.73)	101 (49.27)	0.18
No	37 (29.60)	88 (70.40)		54 (43.20)	71 (56.80)	
**Vaccinated against COVID-19**						
Yes	87 (41.63)	122 (58.37)	0.73	108 (51.67)	101 (48.33)	0.07
No	48 (39.67)	73 (60.33)		50 (41.32)	71 (58.68)	
**Received seasonal influenza vaccine**						
Yes	8 (33.33)	16 (66.67)	0.43	12 (50.00)	12 (50.00)	0.83
No	127 (41.50)	179 (58.50)		146 (47.71)	160 (52.29)	
**History of chickenpox disease**						
Yes	87 (43.28)	114 (56.72)	0.27	110 (54.73)	91 (45.27)	0.0019
No	48 (37.21)	81 (62.79)		48 (37.21)	81 (62.79)	
**Received training programs about Mpox**						
Yes	14 (51.85)	13 (48.15)	0.23	11 (40.74)	16 (59.26)	0.44
No	121 (39.93)	182 (60.07)		147 (48.51)	156 (51.49)	
**Sources of Mpox knowledge**						
Family members	21 (39.62)	32 (60.38)	0.015	30 (56.60)	23 (43.40)	0.39
Friends	4 (13.30)	26 (86.67)		17 (56.67)	13 (43.40)	
Social Medica	50 (40.98)	72 (59.02)		57 (46.72)	65 (53.28)	
Research articles	21 (44.68)	26 (55.32)		22 (46.81)	25 (53.19)	
Scientific websites	39 (50)	39 (50)		32 (41.03)	46 (58.97)	
**Total**	**135 (40.91)**	**195 (59.09)**		**158 (47.88)**	**172 (52.12)**	
*Two-tailed Mann-Whitney u test						

### Predictors of knowledge and attitude

In the knowledge section, students who did not have prior knowledge about smallpox (aOR=0.5, 95% CI=[0.3–0.84]) and those who relied on friends as a source of Mpox information (aOR=0.27, 95% CI=[0.08–0.92]) had significantly lower odds of possessing good knowledge about Mpox. Other variables such as age, gender, stage, and place of residence showed no significant statistical association with knowledge. Regarding attitude, female students had significantly lower odds of a positive attitude compared to males (aOR=0.54, 95% CI=[0.34–0.85]). However, this association was no longer statistically significant in the sensitivity analyses, but the overall trend remained unchanged (Supplementary Material 1). Additionally, students without a history of chickenpox were less likely to have a positive attitude (aOR=0.49, 95% CI=[0.30–0.81]). Other variables in the attitude section missed the significance. Table 4 presents full details of the multivariable binary logistic regression analysis.

**Table 4:** Determinants of medical students’ knowledge and attitude toward human Mpox based on a multivariable binary logistic regression model (n = 330).

Variables	Knowledge	Attitude
	Adjusted odds ratio [95% CI]	Adjusted odds ratio [95% CI]
**Age (Years)**		
21 and below	Reference	Reference
22 and above	1.3 [0.73-2.3]	0.93 [0.53-1.63]
**Gender**		
Male	Reference	Reference
Female	1.15 [0.72-1.83]	0.54 [0.34-0.85]*
**Stage**		
Pre-clinical stages	Reference	Reference
Clinical stages	1.1 [0.59-2.06]	1.17 [0.64-2.16]
**Place of Residence**		
Urban/City	Reference	Reference
Rural	0.83 [0.35-1.95]	0.49 [0.20-1.20]
**Knowledge of smallpox**		
Yes	Reference	Reference
No	0.5 [0.30-0.84]*	0.93 [0.56-1.56]
**Vaccinated against COVID-19**		
Yes	Reference	Reference
No	0.94 [0.58-1.54]	0.71 [0.44-1.15]
**Received seasonal influenza vaccine**		
Yes	Reference	Reference
No	1.48 [0.60-3.72]	1.13 [0.47-2.71]
**History of chickenpox disease**		
Yes	Reference	Reference
No	0.97 [0.58-1.60]	0.49 [0.30-0.81]*
**Received training programs about Mpox**		
Yes	Reference	Reference
No	0.80 [0.34-1.87]	1.69 [0.71-4.0]
**Sources of Mpox knowledge**		
Family members	Reference	Reference
Friends	0.27 [0.08-0.92]*	0.93 [0.36-2.44]
Social media	1.14 [0.57-2.26]	0.62 [0.31-1.23]
Research articles	1.41 [0.60-3.32]	0.79 [0.34-1.86]
Scientific websites	1.65 [0.78-3.47]	0.57 [0.27-1.22]

*p-value <0.05.

## Discussion

In recent decades, globalization has significantly contributed to the spread of infectious diseases, posing a major public health concern worldwide due to the potential rapid spread of pathogens from endemic to non-endemic regions [20]. In the present study, about three-fifths of the students had poor knowledge about human Mpox. The multivariable binary logistic regression model revealed a significant association between poor knowledge of Mpox and those who had inadequate knowledge of smallpox, as well as students who relied on friends as a source of knowledge. However, study participants demonstrated a better attitude, with one in every two students having a positive attitude. Factors like male gender and those with a previous history of chickenpox showed a statistically significant association with a positive attitude.

In the current study, only (8.18%) of the participants have received a training program about Mpox infection. Our findings align with a study carried out in 27 countries, which reported that only (12.5%) of their students have received a training program [11]. The primary sources of Mpox knowledge among our students were social media (36.9%). Similar findings were reported in studies conducted in neighboring countries, including Iran, Saudi Arabia, and the United Arab Emirates (UAE) [18,20,21]. Social media, although an essential tool for disseminating knowledge, often becomes a channel through which unverified, biased, and anecdotal information spreads. This raises serious concerns regarding the potential spread of myths and misinformation within the community.

This concern is not unique to Mpox and has been observed during previous public health emergencies. The COVID-19 pandemic exemplifies how social media can significantly contribute to the spread of falsehoods. This spread of false knowledge results in adverse public attitude that ultimately weaken public trust in health authorities [22]. Misconceptions and false beliefs pose a significant barrier to public health initiatives. Medical students positively influence public perceptions of diseases. Therefore, all students must be equipped with adequate knowledge concerning these re-emerging infectious diseases. Since, inappropriate knowledge and reliance on unauthorized sources for knowledge acquisition among students may result in the dissemination of false beliefs within the general population [23].

In this context, the study’s participants had relatively poor knowledge regarding the Mpox. Nearly three-fifths (59.09%) of participants showed limited knowledge regarding Mpox infection. However, studies carried out among medical students in Saudi Arabia and undergraduate medical and pharmacy students in Vietnam found that (72%) and (65.8%) of their students have poor knowledge about the Mpox, respectively [24,25]. Surprisingly, a study in the UAE revealed that only one-fifth of their students had poor knowledge [21]. This difference can be explained by that approximately two-thirds of students in the UAE study have received information about human Mpox during their academic years. Whereas, in the current study, less than (10%) of students have received a training program during their education. Notably, about two-thirds of the study participants expressed the need to further enhance their knowledge regarding the Mpox. This promising finding will encourage them to participate in Mpox educational initiatives and campaigns in the future.

In addition to medical students and health sciences students, studies have also reported knowledge gaps among HCWs and the public in various regions of the world, including developed countries such as the United States of America and Italy [26–30]. Owing to widespread knowledge gaps across different targeted groups globally, international collaborations are vital to enhance awareness and improve preparedness. This could include organization of training sessions for health sciences students, HCWs, and the broader public. In light of these global knowledge gaps, the present study found that approximately three-quarters of students were unaware of the availability of an effective vaccine against Mpox. Moreover, about two-fifths believed that the recent Mpox outbreak was linked to homosexuality. This combination of low awareness and stigmatizing misconception can create a dual barrier to vaccination programs. Consequently, it may lead to inadequate vaccine coverage, and continued outbreaks among vulnerable groups. A systematic review and meta-analysis reported moderate Mpox vaccine acceptance among students [31].

Several sociocultural norms, pattern of media consumption, and gaps within medical curriculum may have contributed to pervasiveness of these misconceptions. In our region, medical colleges often give limited attention to emerging infectious diseases that are not locally endemic, potentially resulting in insufficient evidence-based knowledge among students. As a result, many students rely on social media as their primary source of information, where content may be fragmented and lack scientific nuance. Furthermore, during early Mpox outbreak in 2022, the international media framed Mpox outbreaks as disproportionately affecting homosexual men, which may have shaped both students and public perceptions [32]. Given that discussion about sexual topics remain socially and culturally sensitive in our setting, such sociocultural norms combined with gaps in medical curriculum had resulted in circulation of these misconceptions [33].

Medical colleges and health institutions should address these gaps by incorporating evidenced-based training on emerging infectious disease, tailored to the local context and students’ needs. Additionally, public health initiatives should adopt culturally and religiously acceptable strategies to correct misinformation and decrease vaccine hesitancy. Further studies exploring cultural and regional factors influencing vaccine hesitancy and acceptance are recommended.

Moreover, apart from knowledge gaps, the overall attitude score of the study participants cannot be considered highly satisfactory. Approximately half of the respondents demonstrated a positive attitude. Comparable results were documented in a study among Egyptian medical students and HCWs, where (44.5%) had a positive attitude [34]. While a study conducted among HCWs in Ethiopia revealed that (62%) of its participants have a positive attitude. In the present study, about half of the students worried that Mpox would be transmitted to their region. These worrying attitudes were also reported among medical students and HCWs in 11 Arabic countries [35]. In Pakistan, approximately half of their general population believed that Mpox would cause a pandemic similar to COVID-19 [36]. However, our participants demonstrated a better attitude regarding this statement.

Additionally, our study demonstrated that older age students had significantly higher knowledge score. However, this association was attenuated and became non-significant in the multivariable logistic regression model, suggesting that variables such as smallpox knowledge and/or information sources may have confounded or mediated this relationship. In support of this, students with prior knowledge of smallpox showed a significantly higher knowledge score. Whereas those who relied on friends as their main sources of information had lower odds of having a good knowledge.

Since, Mpox and smallpox belong to the *Orthopoxvirus* genus within the *Poxviridae* family, it is reasonable that students with a previous background on smallpox to have better comprehension and understanding of closely related viruses [1]. In Vietnam, medical and pharmacy students who utilized scientific websites for information regarding Mpox demonstrated higher levels of knowledge [25]. Also, in the UAE, older students, females, those with a history of chickenpox infection, and received Mpox training significantly exhibited a higher level of knowledge [21].

With respect to attitudes, male students expressed more positive attitudes toward Mpox. Given that males were disproportionately affected during the recent global Mpox outbreak, this may have enhanced male students’ awareness and preparedness toward this emerging infectious disease [37]. This finding aligns with an Egyptian study carried out among HCWs and medical students, which also revealed that male gender was associated with a more positive attitude compared to their peers [34]. Contrary to our findings, a study found that male students were more likely to have a negative attitude [11].

Furthermore, this significant positive correlation is supported by evidence from a regional study, which reported a higher level of worry about Mpox among males; and the media has been considered as one of the primary sources contributing to this worry [26]. Additionally, a study conducted in Iraq found that males demonstrated higher health seeking behavior compared to females [38]. This gender disparity in health seeking behavior in our region could be explained by several sociocultural barriers. In Iraq, public perceptions and misunderstanding regarding sexual and reproductive health issues often discourage women from seeking healthcare. Moreover, due to conservative social norms women are less likely to be employed, limiting their exposure to health information’s. Finally, a lower percentage of woman complete secondary education compared to males [39].

However, the proposed explanation for the observed gender disparity in the attitude section remains speculative. This is because our study was not designed to investigate the underlying determinants of this difference, and no variables related to sociocultural factors and health-seeking behavior were assessed. Furthermore, the current dataset provides no empirical evidence to support these interpretations, and the association lost statistical significance in sensitivity analyses. Therefore, in the absence of supporting data, these explanations remain hypotheses requiring further testing rather than evidence-based conclusions, and thus, it should be interpreted with caution.

Furthermore, our findings suggest that previous exposure to chickenpox was significantly associated with a more positive attitude. This is likely because prior experience with viral exanthem enhances individual adherence to preventive and control measures and may enhance their general interest and awareness regarding infectious diseases, making them more interested in infectious diseases information. These disparities in knowledge and attitude level across studies may be attributed to differences in cultural perceptions, access to information, variation in educational systems, and different regional responses to infectious disease outbreaks.

### Strengths and limitations of the study

The strength of this study lies in being the first study to assess medical students’ knowledge and attitudes toward Mpox in the Kurdistan Region of Iraq, and application of sensitivity analyses, which ensured robustness and consistency of the observed association using various analytical approaches. Despite this, our study poses some limitations that need exploration. First, the study utilized a self-reporting survey, which may introduce erroneous information, response bias, and self-selection. Secondly, despite using an anonymous data collection method, the potential for social desirability bias in response to the attitude section remains. Additionally, the study questionnaire demonstrated acceptable, but borderline internal consistency, and since the study included students from only two medical schools in Iraqi Kurdistan, these factors limit the generalizability of the findings to all medical and health sciences students in the Kurdistan Region of Iraq. Therefore, the results should be interpreted and used in caution, especially when applied to the broader regional students or when utilizing subgroup analysis, as the data are more suitable for descriptive purposes rather than comparative analysis. Nonetheless, we believe that the study will play a critical role in shaping future research efforts about Mpox and emerging infectious diseases among medical students in our region. Finally, the cross-sectional design of the study limits our ability to conclude a cause-and-effect relationship.

## Conclusions and recommendations

Medical students in the present study demonstrated relatively insufficient knowledge about Mpox. However, their attitudes were more favorable, albeit still suboptimal. Factors such as older age, and knowledge about smallpox were significantly associated with better knowledge, whereas students who relied on friends as a primary source of information demonstrated significantly lower knowledge. Additionally, male students, and those with a history of chickenpox infection had a more positive attitude.

These findings highlight the urgent necessity for structured educational programs on emerging infectious diseases targeting health sciences students, HCWs, and the general public. In addition, medical colleges should consider integrating Mpox-related lectures into their curricula, with particular emphasis on routes of transmission, necessity of vaccination, and strategies to reduce stigma and correct misconceptions surrounding Mpox. Such initiatives are essential to equip students with the necessary knowledge and skills to effectively respond to and control infectious disease outbreaks, ultimately strengthening public health. In the present study, only a small proportion of students received training on Mpox, which limits our ability to meaningfully assess effectiveness of such training programs within this sample. However, a previous study has demonstrated that educational interventions assessed through pre-test and post-test design have substantially increased students’ knowledge and attitudes regarding Mpox infection [40]. Hence, we recommend that the impact of these educational initiatives should be evaluated by further studies to assess their effect on student awareness and preparedness in our region.

Social media appeared as a primary source of information regarding Mpox. Medical institutions and health ministry should take opportunities from these platforms to disseminate evidence-based information through collaboration with popular medical educators to create and share engaging infographic-based content regarding Mpox transmission and prevention. This will reduce misconceptions and increase public awareness.

### Declaration of generative AI and AI-assisted technologies in the writing process

During the preparation of this manuscript, the authors used ChatGPT 4o in order to improve the grammar and linguistic format of the manuscript. After using this tool, the authors reviewed and edited the content as needed and take full responsibility for the content of the published article.

## Supporting information

S1 File Sensitivity analyses. (DOCX)

S2 Table Medical students’ knowledge and attitude toward human Mpox. Contains Table 2A and Table 2B.
(DOCX)

S3 Data Minimal dataset. (XLSX)
