# Supplementary material for: Determinants of knowledge and attitudes toward Mpox among medical students: A cross-sectional study from Kurdistan region of Iraq
Source: PLoS One. 2026 May 29;21(5):e0350502. doi: 10.1371/journal.pone.0350502 (PMC13221006; doi:10.1371/journal.pone.0350502)
Supplement: S1 File — (DOCX) [file pone.0350502.s001.docx]

**Supplementary Material 1**

**Sensitivity analyses methods and interpretation**

Sensitivity analyses were performed to assess robustness of the findings by modeling dependent variables as continuous measures and by applying alternative cut-off criteria. When knowledge and attitude scores were analyzed as continuous variables, non-parametric tests were employed due to non-normal distribution of data. Specifically, the two-tailed Mann-Whitney test was used when comparing two independent groups, while the Kruskal-Wallis H test was utilized for comparisons between more than two independent samples. In this model, all previously identified significant predictors using different cut-off value remained statistically significant, expect for gender in the attitude section, which lost statistical significance. However, the overall trend showing that male students have better attitude remained similar and didn’t change. **Table 1A** shows complete results of sensitivity analysis using continuous score modeling.

Furthermore, sensitivity analysis using Bloom’s criteria as a cut-off point was also performed, in which scores ≥ 80% were classified as high, 79%-60% as intermediate, and <60% as low. The statistical significance between independent and dependent variables was established using Pearson Chi-square. Under this categorization, all significant determinants in the knowledge section remained unchanged (**Table 1B**). In contrast, within the attitude section, the association between history of chickenpox infection and Mpox attitude just lost statistical significance (p=0.06) (**Table 1C**). Nevertheless, the trend that students with history of chickenpox infection had better attitude remained consistent.

| **Table 1A. Sensitivity analysis of predictors of knowledge and attitude using continuous score modeling** | | | | |
| --- | --- | --- | --- | --- |
| **Variables** | **Knowledge**  **Median [IQR]** | **p-value** | **Attitude**  **Median [IQR]** | **p-value** |
| **Age (Year)** |  |  |  |  |
| 21 and below | 19 [16-22] | 0.016 | 42 [36-45] | 0.38 |
| 22 and above | 20 [17-24] |  | 42 [38-45] |  |
| **Gender** |  |  |  |  |
| Male | 20 [16-22] | 0.39 | 43 [38-46] | 0.072 |
| Female | 20 [16-23] |  | 41.5 [36-45] |  |
| **Stage** |  |  |  |  |
| Pre-clinical stages | 20 [15-22] | 0.02 | 41 [36-45] | 0.055 |
| Clinical stages | 20 [17-23] |  | 43 [39-46] |  |
| **Place of Residence** |  |  |  |  |
| Urban/City | 20 [16-23] | 0.34 | 42 [37-45] | 0.22 |
| Rural | 20 [16.75-25] |  | 40 [34.5-45] |  |
| **Knowledge of smallpox** |  |  |  |  |
| Yes | 20 [17-24] | <0.001 | 43 [37-46] | 0.27 |
| No | 18 [14-21] |  | 42 [37-45] |  |
| **Vaccinated against COVID-19** |  |  |  |  |
| Yes | 20 [16.5-23] | 0.45 | 43 [37-46] | 0.098 |
| No | 20 [16-23] |  | 41 [37-45] |  |
| **Received seasonal influenza vaccine** |  |  |  |  |
| Yes | 20 [19-22] | 0.86 | 42 [36-44.75] | 0.74 |
| No | 20 [16-23] |  | 42 [37-45.25] |  |
| **History of chickenpox disease** |  |  |  |  |
| Yes | 20 [17-23] | 0.18 | 43 [39-46] | 0.001 |
| No | 19 [15-23] |  | 40 [36-45] |  |
| **Received training programs about Mpox** |  |  |  |  |
| Yes | 22 [17-24] | 0.12 | 40 [36-45] | 0.22 |
| No | 20 [16-23] |  | 42 [37-45] |  |
| **Sources of Mpox knowledge** |  |  |  |  |
| Family members | 18 [16-23] | 0.028^#^ | 44 [37-46.5] | 0.49^#^ |
| Friends | 17 [15-20] |  | 43 [38.5-46] |  |
| Social Medica | 20 [16-23] |  | 42 [37-45] |  |
| Research articles | 20 [18-23] |  | 42 [36-45] |  |
| Scientific websites | 20.5 [17-24] |  | 42 [35.75-45] |  |
| #Kruskal-Wallis H test | | | | |

| **Table 1B. Sensitivity analysis of determinants of knowledge using Bloom’s criteria as a cut-off point (n=330)** | | | | |
| --- | --- | --- | --- | --- |
| **Variables** | **Knowledge** | | | **p-value** |
|  | **High (≥80%)**  **n (%)** | **Moderate (79%-60%)**  **n (%)** | **Low (<60%)**  **n (%)** |  |
| **Age (Year)** |  |  |  |  |
| 21 and below | 37 (23.13) | 71 (44.37) | 52 (32.5) | 0.021 |
| 22 and above | 59 (34.70) | 75 (44.12) | 36 (21.18) |  |
| **Gender** |  |  |  |  |
| Male | 37 (23.42) | 80 (50.63) | 41 (25.95) | 0.045 |
| Female | 59 (34.30) | 66 (38.37) | 47 (27.33) |  |
| **Stage** |  |  |  |  |
| Pre-clinical stages | 28 (22.05) | 57 (44.88) | 42 (33.07) | 0.035 |
| Clinical stages | 68 (33.50) | 89 (43.84) | 46 (22.66) |  |
| **Place of Residence** |  |  |  |  |
| Urban/City | 86 (28.29) | 136 (44.74) | 82 (26.97) | 0.55 |
| Rural | 10 (38.46) | 10 (38.46) | 6 (23.08) |  |
| **Knowledge of smallpox** |  |  |  |  |
| Yes | 75 (36.59) | 91 (44.39) | 39 (19.02) | <0.001 |
| No | 21 (16.80) | 55 (44) | 49 (39.20) |  |
| **Vaccinated against COVID-19** |  |  |  |  |
| Yes | 63 (30.14) | 94 (44.98) | 52 (24.88) | 0.62 |
| No | 33 (27.27) | 52 (42.98) | 36 (29.75) |  |
| **Received seasonal influenza vaccine** |  |  |  |  |
| Yes | 4 (16.67) | 16 (66.66) | 4 (16.67) | 0.071 |
| No | 92 (30.07) | 130 (42.48) | 84 (27.45) |  |
| **History of chickenpox disease** |  |  |  |  |
| Yes | 58 (28.86) | 96 (47.76) | 47 (23.38) | 0.17 |
| No | 38 (29.46) | 50 (38.76) | 41 (31.78) |  |
| **Received training programs about Mpox** |  |  |  |  |
| Yes | 11 (40.74) | 12 (44.44) | 4 (14.82) | 0.23 |
| No | 85 (28.05) | 134 (44.23) | 84 (27.72) |  |
| **Sources of Mpox knowledge** |  |  |  |  |
| Family members | 15 (28.30) | 24 (45.28) | 14 (26.42) | 0.036 |
| Friends | 1 (3.34) | 19 (63.33) | 10 (33.33) |  |
| Social Medica | 36 (29.51) | 51 (41.80) | 35 (28.69) |  |
| Research articles | 12 (25.53) | 24 (51.06) | 11 (23.41) |  |
| Scientific websites | 32 (41.03) | 28 (35.90) | 18 (23.07) |  |
| **Total** | **96 (29.09)** | **146 (44.24)** | **88 (26.67)** |  |

| **Table 1C. Sensitivity analysis of determinants of attitudes using Bloom’s criteria as a cut-off point (n=330)** | | | | |
| --- | --- | --- | --- | --- |
| **Variables** | **Attitude** | | | **p-value** |
|  | **Positive (≥80%)**  **n (%)** | **Neutral (79%-60%)**  **n (%)** | **Negative (<60%)**  **n (%)** |  |
| **Age (Year)** |  |  |  |  |
| 21 and below | 19 (11.88) | 107 (66.87) | 34 (21.25) | 0.34 |
| 22 and above | 19 (11.18) | 125 (73.53) | 26 (15.29) |  |
| **Gender** |  |  |  |  |
| Male | 19 (12.02) | 113 (71.52) | 26 (16.46) | 0.73 |
| Female | 19 (11.05) | 119 (69.18) | 34 (19.77) |  |
| **Stage** |  |  |  |  |
| Pre-clinical stages | 13 (10.24) | 87 (68.50) | 27 (21.26) | 0.48 |
| Clinical stages | 25 (12.31) | 145 (71.43) | 33 (16.26) |  |
| **Place of Residence** |  |  |  |  |
| Urban/City | 33 (10.86) | 218 (71.71) | 53 (17.43) | 0.16 |
| Rural | 5 (19.23) | 14 (53.85) | 7 (26.92) |  |
| **Knowledge of smallpox** |  |  |  |  |
| Yes | 27 (13.17) | 141 (68.78) | 37 (18.05) | 0.48 |
| No | 11 (8.8) | 91 (72.80) | 23 (18.4) |  |
| **Vaccinated against COVID-19** |  |  |  |  |
| Yes | 27 (12.92) | 146 (69.86) | 36 (17.22) | 0.53 |
| No | 11 (9.09) | 86 (71.07) | 24 (19.84) |  |
| **Received seasonal influenza vaccine** |  |  |  |  |
| Yes | 2 (8.33) | 18 (75.00) | 4 (16.67) | 0.84 |
| No | 36 (11.77) | 214 (69.93) | 56 (18.30) |  |
| **History of chickenpox disease** |  |  |  |  |
| Yes | 28 (13.93) | 143 (71.14) | 30 (14.93) | 0.06 |
| No | 10 (7.75) | 89 (68.99) | 30 (23.26) |  |
| **Received training programs about Mpox** |  |  |  |  |
| Yes | 3 (11.11) | 18 (66.67) | 6 (22.22) | 0.85 |
| No | 35 (11.55) | 214 (70.63) | 54 (17.82) |  |
| **Sources of Mpox knowledge** |  |  |  |  |
| Family members | 8 (15.09) | 36 (67.93) | 9 (16.98) | 0.59 |
| Friends | 3 (10.00) | 25 (83.33) | 2 (6.67) |  |
| Social Medica | 12 (9.84) | 89 (72.95) | 21 (17.21) |  |
| Research articles | 5 (10.64) | 33 (70.21) | 9 (19.15) |  |
| Scientific websites | 10 (12.82) | 49 (62.82) | 19 (24.36) |  |
| **Total** | **38 (11.52)** | **232 (70.30)** | **60 (18.18)** |  |
