# Supplementary material for: Determinants of knowledge and attitudes toward Mpox among medical students: A cross-sectional study from Kurdistan region of Iraq
Source: PLoS One. 2026 May 29;21(5):e0350502. doi: 10.1371/journal.pone.0350502 (PMC13221006; doi:10.1371/journal.pone.0350502)
Supplement: S2 Table — Contains Table 2A and Table 2B. (DOCX) [file pone.0350502.s002.docx]

**Supplementary Material 2**

| **Table 2A. Knowledge of medical students about Mpox infection (n=330)** | | | |
| --- | --- | --- | --- |
| **Variables** | **Yes n (%)** | **Uncertain n (%)** | **No n (%)** |
| 1. Human Mpox is a viral disease | 249 (75.45) | 71 (21.52) | 10 (3.03) |
| 2. Mpox is a re-emerging disease | 125 (37.88) | 153 (46.36) | 52 (15.76) |
| 3. Mpox is easily transmitted from animal-to-human through direct contact | 163 (49.39) | 128 (38.79) | 39 (11.82) |
| 4. Blood-borne transmission of Mpox is possible | 184 (55.76) | 125 (37.88) | 21 (6.36) |
| 5. Mpox can be transmitted through eating contaminated meat | 104 (31.52) | 147 (44.55) | 79 (23.94) |
| 6. Mpox outbreaks in 2022 were noted to be related to homosexuality | 127 (38.48) | 180 (54.55) | 23 (6.97) |
| 7. Skin rashes are one of clinical manifestations of Mpox disease | 240 (72.73) | 76 (23.03) | 14 (4.24) |
| 8. Avoiding contact with wild animals (alive or dead) is essential to prevent further Mpox transmission | 202 (61.21) | 112 (33.94) | 16 (4.85) |
| 9. Mpox could be prevented by cooking meat properly | 145 (43.94) | 143 (43.3) | 42 (12.73) |
| 10. Avoiding contact with any objects that have been in contact with sick animal can prevent spread of disease | 208 (63.03) | 94 (28.48) | 28 (8.48) |
| 11. Avoiding contact with person that has a rash can prevent the spread of disease | 211 (63.94) | 89 (26.97) | 30 (9.09) |
| 12. Avoiding contact with any object that has been in contact with sick person can prevent spread of disease | 196 (59.39) | 99 (30) | 35 (10.61) |
| 13. Reporting Mpox signs to local health authorities is essential to prevent further disease transmission | 238 (72.12) | 79 (23.94) | 13 (3.94) |
| 14. There was a licensed Mpox vaccine available at the time of this study | 85 (25.76) | 206 (62.42) | 39 (11.82) |

| **Table 2B. Medical students’ attitudes toward Mpox infection (n=330)** | | | | | |
| --- | --- | --- | --- | --- | --- |
| **Variables** | **Strongly Agree**  **n (%)** | **Agree**  **n (%)** | **Neutral**  **n (%)** | **Disagree**  **n (%)** | **Strongly Disagree**  **n (%)** |
| 1. I should learn more about Mpox disease | 149 (45.15) | 70 (21.21) | 64 (19.39) | 19 (5.77) | 28 (8.48) |
| 2. I worry that Mpox disease can be transmitted to my country | 73 (22.12) | 87 (26.36) | 114 (34.55) | 35 (10.61) | 21 (6.36) |
| 3. Mpox disease prevention and control measures should be adequately available | 151 (45.76) | 88 (26.67) | 50 (15.15) | 24 (7.27) | 17 (5.15) |
| 4. Traveling to Mpox disease-infected countries should be restricted | 107 (32.42) | 80 (24.24) | 91 (27.58) | 33 (10) | 19 (5.76) |
| 5. I should take Mpox vaccine if it is available | 108 (32.73) | 75 (22.73) | 91 (27.58) | 28 (8.48) | 28 (8.48) |
| 6. Health care workers should be tested when they are in contact with someone infected | 152 (46.06) | 80 (24.24) | 55 (16.67) | 22 (6.67) | 21 (6.36) |
| 7. I can visit any family members or friends who are infected with Mpox | 31 (9.39) | 31 (9.39) | 70 (21.22) | 82 (24.85) | 116 (35.15) |
| 8. I should take more hygienic preventive measures due to Mpox disease | 109 (33.03) | 94 (28.48) | 89 (26.98) | 18 (5.45) | 20 (6.06) |
| 9. All people with a skin rash should be tested for Mpox | 41 (12.42) | 79 (23.94) | 109 (33.03) | 68 (20.61) | 33 (10) |
| 10. I worry that Mpox will become a new pandemic, and its impact will be like COVID-19 | 38 (11.52) | 64 (19.39) | 107 (32.42) | 79 (23.94) | 42 (12.73) |
| 11. I do not trust the information about diseases from scientific experts | 24 (7.27) | 24 (7.27) | 88 (26.67) | 91 (27.58) | 103 (31.21) |
| 12. I worry that Mpox disease is an attempt to reduce the size of global population | 27 (8.18) | 37 (11.21) | 92 (27.88) | 69 (20.91) | 105 (31.82) |
